# Accelerating Multi-Objective Optimization of Composite Structures Using Multi-Fidelity Surrogate Models and Curriculum Learning

**DOI:** 10.3390/ma18071469

**Published:** 2025-03-26

**Authors:** Bartosz Miller, Leonard Ziemiański

**Affiliations:** Faculty of Civil and Environmental Engineering and Architecture, Rzeszów University of Technology, Al. Powstancow Warszawy 12, 35-959 Rzeszow, Poland; bartosz.miller@prz.edu.pl

**Keywords:** multi-objective optimization, composite, multi-fidelity models, surrogate models, deep neural networks, genetic algorithms, curriculum learning

## Abstract

The optimization of multilayer composite structures requires balancing mechanical performance, economic efficiency, and computational feasibility. This study introduces an innovative approach that integrates Curriculum Learning (CL) with a multi-fidelity surrogate model to enhance computational efficiency in engineering design. A multi-fidelity strategy is introduced to generate training data efficiently, leveraging a high-fidelity finite element model for accurate simulations and a low-fidelity model to provide a larger dataset at reduced computational cost. Unlike conventional surrogate modeling approaches, the proposed method applies CL to iteratively refine the surrogate model, enabling step-by-step learning of complex structural patterns and improving prediction accuracy. Genetic algorithms (GAs) are then applied to optimize structural parameters while minimizing computational expense. The integration of CL and multi-fidelity modeling allows for a reduction in computational burden while preserving accuracy, demonstrating practical applicability in real-world structural design problems. The effectiveness of this methodology is validated by evaluating Pareto front quality using selected performance indicators. Results demonstrate that the proposed approach reduces optimization burden while achieving accurate predictions, highlighting the benefits of integrating surrogate modeling, multi-fidelity analysis, CL, and GAs for efficient composite structure optimization. This work contributes to the advancement of optimization methodologies by providing a scalable framework applicable to complex engineering problems requiring high computational efficiency.

## 1. Introduction

Multilayer composite structures are widely used in various industries, including aerospace, automotive, and construction, where the optimization of parameters such as load-bearing capacity, fatigue resistance, and vibration damping is essential [1,2,3,4]. The design of multilayer composite structures poses a significant engineering challenge, particularly in the selection of materials with different mechanical properties and economic factors [5]. This process requires precise selection of materials for individual layers so that the resulting structure meets specified strength, technological, and operational requirements while minimizing costs. The complexity of this issue makes classical single-optimization methods insufficient, leading to the necessity of applying a multi-criteria approach that allows the simultaneous analysis of multiple design aspects, including both mechanical and economic parameters.

One of the effective tools for solving complex optimization problems is the application of genetic algorithms (GAs) [6,7,8,9]. These methods enable the efficient exploration of the solution space to minimize the objective function while considering both mechanical and economic constraints. Genetic algorithms, inspired by evolutionary processes, allow for the iterative improvement of solutions through selection, crossover, and mutation of a population of solutions. However, the use of GAs involves repeated objective function evaluations, which, in the case of advanced analyses based on the Finite Element Method (FEM), leads to high computational costs.

To reduce computational time, surrogate models are used to quickly approximate FEM analysis results [10,11,12]. A key challenge is building a sufficient dataset to train the surrogate model. A multi-fidelity (MF) approach is often applied in this process, utilizing FEM models with varying levels of accuracy [13]. Lower-resolution numerical models (low-fidelity, LF) enable fast data generation but require correction techniques to improve their reliability. Various methods exist for integrating results obtained from models of different fidelity levels, including statistical methods and machine learning-based algorithms. The choice of an appropriate strategy affects both the training time and the accuracy of the surrogate model.

Different methods, such as Kriging and co-Kriging, statistical models, and deep neural networks, are commonly used to develop surrogate models [11,12,14,15,16,17,18,19,20]. Each of these approaches offers varying levels of accuracy and computational complexity, making it crucial not only to assess the effectiveness of the surrogate model itself but also its impact on optimization results. Kriging, as one of the widely used interpolation methods, enables the precise modeling of nonlinear relationships between input parameters and FEM analysis results. On the other hand, deep neural networks can model highly complex nonlinear dependencies but require substantial computational resources and large training datasets [21,22,23].

The concept of Curriculum Learning (CL) originates from human cognition, where learning involves acquiring knowledge through exposure to successive samples in a structured sequence—progressing from the simplest to the most complex examples. This idea was first introduced into machine learning by Bengio et al. [24] in 2009, who suggested that such an approach not only accelerates the training process but also improves the quality of the local minima obtained. In subsequent years, the concept of progressive learning was applied across various domains with numerous modifications and extensions. Despite these differences, a common feature of these approaches is the emphasis on refining models by focusing on increasingly challenging or problematic examples. For instance, Hsiao and Chang [25] utilized CL for constructing surrogate models to describe chemical processes (namely, an amine scrubbing process), demonstrating its effectiveness in improving model accuracy.

The concept has also been extended to continual learning, where models are incrementally trained on new data while retaining previously acquired knowledge, as explored by Fayek et al. [26]. Another advanced adaptation, referred to as adaptive continual learning, was proposed by Kong et al. [27], in which each learning step was dynamically adjusted based on results obtained in preceding steps, further enhancing model performance. A notable application of CL to highly complex systems includes its use in modeling unsteady hypersonic flows in chemical nonequilibrium, as demonstrated by Scherding et al. [28]. This study highlights the potential of curriculum-based approaches in computationally demanding simulations, reinforcing the broader utility of this learning paradigm across various scientific and engineering disciplines.

The effectiveness of CL has been demonstrated across various complex applications, where structured, progressive training significantly enhances model performance. In many challenging problems, the approach described in this study as CL provides substantial improvements in result quality while simultaneously reducing computational effort, making it a valuable tool for optimizing surrogate modeling and numerical simulations.

The verification of solution quality cannot rely solely on comparing the surrogate model with FEM results but must also consider the correctness of the optimization at a global level. This necessitates evaluating the influence of the surrogate model on results obtained using GA-based optimization. Furthermore, the optimization process must account for modeling uncertainties, which may require applying probabilistic methods for result analysis.

Due to the multi-criteria nature of the problem and the need for a continuous comparison of optimization results, appropriate indicators are used to assess solution quality [29,30]. One of the widely used tools is the analysis of Pareto fronts, enabling the evaluation of trade-offs between different optimization criteria. The Pareto front identifies a set of non-dominated solutions, where each represents an optimal compromise between multiple optimization objectives. This allows for determining optimal material configurations and evaluating their effectiveness concerning predefined design criteria.

Addressing these challenges is a key aspect of effective composite structure optimization, enabling the development of design methodologies that provide optimal solutions both in technical and economic terms. By integrating computational methods, optimization algorithms, and machine learning techniques, it is possible to create more efficient tools to support the design process of multilayer composites. Modern optimization approaches also consider sustainability and environmental constraints, which can serve as additional factors in the design analysis.

Queipo et al. [31] explored surrogate-based methods for analysis and optimization, addressing key aspects such as loss function selection, regularization criteria, experimental design strategies, sensitivity analysis, and convergence assessment. Their study also illustrated state-of-the-art applications through a multi-objective optimization case involving a liquid rocket injector.

In their comprehensive review, Forrester and Keane [12] examined the latest advancements in surrogate model construction and their integration into optimization strategies. Their work provided a detailed evaluation of the advantages and limitations of various surrogate modeling techniques, offering practical guidelines for their implementation. Additionally, Hwang and Martins [32] analyzed the behavior of several popular surrogate modeling approaches when applied to problems requiring thousands of training samples.

The optimization of the dynamic behavior of shell structures has been widely studied, with numerous algorithms proposed in recent research to tackle this challenge. For example, Jing et al. [33] introduced a sequential permutation search algorithm aimed at optimizing the stacking sequence of doubly curved laminated composite shallow shells to maximize the fundamental frequency. Similarly, Chen et al. [34] developed a multivariate improved sparrow search algorithm to enhance the fundamental frequency of composite laminated cylindrical shells while minimizing vibrational resonance. Chaudhuri et al. [35] performed a numerical investigation into the free vibration response of composite stiffened hypar shells with cutouts, utilizing an FE analysis. Their optimization relied on parametric tuning based on the Taguchi approach to achieve the desired frequency response. Another study by Serhat [36] focused on optimizing the eigenfrequencies of circular cylindrical laminates by examining the influence of parameters such as cylinder length, radius, thickness, and boundary conditions. Likewise, Alshabatat [37] explored the optimization of natural frequencies in circular cylindrical shells using axially functionally graded materials. Collectively, these studies contribute to advancing optimization methodologies for improving the dynamic performance of composite structures.

This study aims to address the challenges associated with optimizing the dynamic properties of multilayer composite structures while minimizing computational costs. The proposed methodology integrates MF FE models with deep neural network-based surrogate modeling, enabling efficient and accurate multi-objective optimization.

The novelty of this research lies in the systematic use of surrogate models within a CL framework specifically tailored for multi-objective optimization. Unlike traditional surrogate modeling approaches, where training is performed using a fixed dataset, the proposed method dynamically improves the surrogate model by incorporating new high-fidelity samples in successive CL iterations. This iterative refinement enhances the predictive accuracy of the surrogate model while ensuring better convergence of the optimization process. By progressively increasing the quality of the surrogate model, the CL-based approach enables a more reliable identification of the Pareto front, leading to improved trade-off solutions between competing objectives while maintaining computational efficiency.

Furthermore, this study explores different architectures for the surrogate model, comparing three distinct configurations. The effectiveness of these variants is assessed using Pareto front quality indicators, providing a comprehensive evaluation of their impact on optimization performance.

By incorporating these innovations, the proposed methodology offers a robust and scalable solution for optimizing composite structures, demonstrating its applicability to engineering problems requiring a balance between accuracy and computational feasibility.

## 2. Materials and Methods

### 2.1. Vibration Problem

In dynamic structural analysis, an essential issue is determining the system’s natural frequencies and mode shapes. The equation of motion describing the system’s dynamics can be written as:

(1) $$M\ddot{x}+C\dot{x}+Kx=P$$

where

M is a n×n mass matrix;
C is a n×n damping matrix;
K is a n×n stiffness matrix;
x is a *n*-element vector of nodal displacements;
P is a *n*-element vector of external forces at nodes;
*n* is the number of dynamic degrees of freedom.

For the free-vibration analysis, when external forces are absent and damping is neglected, the equation simplifies to:

(2) $$M\ddot{x}+Kx=0$$

Solving this system leads to the so-called eigenvalue problem:

(3) $$K\Phi =M\Phi \Omega^{2}$$

where Φ represents the matrix of mode shapes $\phi_{i}$ (stored in successive columns of matrix Φ), and Ω is the matrix of eigenvalues $\omega_{i}$. The angular frequencies $\omega_{i}$ divided by 2π yield the natural frequencies $f_{i}$ corresponding to the vibration shapes $\phi_{i}$. Determining the system’s eigenvalues and eigenvectors allows for the analysis of the dynamic properties of the structure, which is crucial for designing and optimizing structures subjected to dynamic excitation.

### 2.2. Analysis of Dynamic Parameters to Avoid the Resonance Phenomenon

In the analysis of structures subjected to dynamic loads, a key aspect is optimizing their dynamic properties to prevent resonance, which can lead to catastrophic consequences. Resonance occurs when the excitation frequency coincides with or is very close to one of the system’s natural frequencies, resulting in a rapid increase in vibration amplitude, which may lead to structural failure. To avoid this, it is necessary to shape the system’s natural frequency spectrum appropriately at the design stage.

If the excitation frequency is known, optimizing the natural frequency spectrum involves maximizing the separation of natural frequencies from this value, creating a frequency gap around the excitation frequency. This approach significantly reduces the risk of resonance. It is also crucial for low-stress structures, where even minor vibrations can cause premature damage or degradation of functional properties.

Shaping the natural frequency spectrum can be performed as part of an optimization procedure with a properly defined objective function. In its basic form, the objective function can be formulated to maximize the distance between the natural frequencies and the excitation frequency:

(4) $$g_{f}\left(p\right)=-\min\left|f\left(p\right)-f_{\mathrm{exc}}\right|,$$

where the vector f(p) gathers the natural frequencies of the investigated model obtained for specific values of design parameters collected in vector p, and $f_{\mathrm{exc}}$ stands for the considered excitation frequency. In this paper, $f_{\mathrm{exc}}=80$ Hz.

If an additional criterion, such as minimizing the structure’s cost, is considered, the optimization problem becomes multi-objective. In this case, the second objective function can be expressed as:

(5) $$g_{c}\left(p\right)=\sum_{i=1}^{8}\left(\frac{V(p)}{8}\cdot c_{i}\right)$$

where V(p) is the total volume of the structure, and $c_{i}$ is the cost per unit volume of the material used in layer *i*.

In this case, the multi-objective optimization aims to minimize both objective functions simultaneously. The standard formulation of the multi-objective optimization problem—finding the values of the arguments collected in an *m*-element structure’s control parameters vector p for which two considered objective functions yield the lowest possible values—is given by:

(6) $$p^{\mathrm{opt}}=\underset{p\in P^{m}}{\arg\min}\left\{g_{f}\left(p\right),g_{c}\left(p\right)\right\},$$

where p is a vector of structure parameters, and $P^{m}$ is the *m*-dimensional space of the decision parameters gathered in vector p.

The solution to the multi-objective optimization problem is the so-called Pareto front. The Pareto front represents the set of non-dominated solutions in a multi-objective optimization problem. A solution is considered non-dominated if no other solution exists that improves one objective without worsening at least one other. In practical applications, the Pareto front provides decision-makers with a range of optimal trade-offs between competing objectives, allowing for a more informed selection of the most suitable design configuration.

To compare results obtained from different optimization approaches, numerical measures of Pareto front quality must be introduced. Pareto front indicators assess the distribution and diversity of solutions. One example is the hypervolume indicator, which measures the volume of space enclosed by the Pareto front concerning a reference point. The greater the value of this indicator, the better the quality of the obtained solutions regarding the distribution of trade-offs among objective functions. Another commonly used metric is the distance of generated solutions from the theoretically optimal Pareto solution, which helps evaluate the accuracy of the optimization process.

Considering these aspects in the design process allows us to obtain a system with optimized dynamic properties while simultaneously minimizing production costs and reducing the risk of damage due to uncontrolled dynamic excitations.

### 2.3. The Analyzed Structure

**Figure 1:**
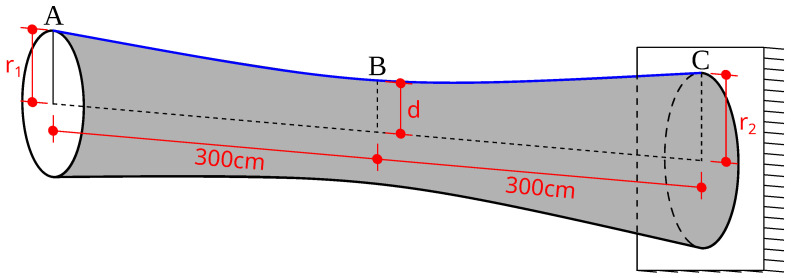
The analyzed structure: cantilever axisymmetric hyperboloid with varying middle-length diameter *d*.

The axisymmetric structure analyzed in this study was generated by rotating a flat hyperbola (marked with blue in Figure 1) around a fixed axis. This hyperbola had predefined fixed start point A and end point C (namely, $r_{1}=61.03$ cm, $r_{2}=73.236$ cm, and the overall length equaled 600 cm), while its middle point B could change its position (given by *d* parameter) along the axis perpendicular to the axis of rotation, allowing control over the shape of the generated shell. This geometry enabled a broad range of structural configurations with varying dynamic and mechanical properties.

The shell was asymmetrically supported—one end was fixed, meaning all degrees of freedom are constrained, while the other end remained free. These boundary conditions led to specific dynamic properties of the structure, directly affecting its natural frequency spectrum and susceptibility to resonance phenomena. The structure is shown in Figure 1.

The analyzed structure was made of a composite material with a constant thickness of 16 mm and consisted of eight layers. Each layer had the same thickness but could be made from one of three available composite materials. Additionally, each layer had a unique fiber orientation, meaning that the orientation of fibers in each layer differed, affecting the mechanical and dynamic properties of the shell.

The complete configuration of the structure was described by the parameter vector p, which consisted of m=17 variables: eight fiber orientation angles $\lambda_{i}$, material selections for each of the eight layers $\mu_{i}$, and one coordinate defining the position of the middle point of the base hyperbola *d*; see Equation (7). This set of parameters allowed for a precise modeling of the shell and its optimization concerning various criteria, including structural dynamics, stiffness, and material and manufacturing costs.

(7) $$\underset{17\times 1}{p}={\left\{\lambda_{1},\lambda_{2},\cdots ,\lambda_{8}\;\mu_{1},\mu_{2},\cdots ,\mu_{8},d\right\}}^{\prime }.$$

The materials used to construct the shell included two real composite materials: Carbon Fiber-Reinforced Polymer (CFRP) and Glass Fiber-Reinforced Polymer (GFRP). Additionally, a theoretical material, the theoretical Fiber-Reinforced Polymer (*t*FRP), was introduced for optimization purposes. The parameters of this material were calculated as the average values of the properties of the CFRP and GFRP. The introduction of this material increased the complexity of the optimization task by introducing an additional value for one of the decision variables.

Table 1 presents a summary of the mechanical and physical properties of the available materials.

### 2.4. Finite Element Models

This study employed two FE models that differed only in their FE size, which effectively means variations in mesh density. Each model consisted of four-node MITC4 multilayered shell elements, which are based on the first-order shear deformation theory [39].

Each layer of the shell structure corresponded to a single composite layer, with potentially different material properties and fiber orientation angles. The maximum side length of an approximately square finite element, denoted as *h*, for the primary FE model (referred to here as M1), was selected to be approximately h=1.25 cm. However, slight variations existed in both the circumferential and longitudinal directions, and also at different locations along the shell’s axis. In addition to the M1 model, one coarse model, labeled as M5, was introduced, featuring element sizes of h=5 cm.

The high-fidelity M1 model served as the basis for constructing a pseudo-experimental model. Meanwhile, the lower-fidelity M5 model contributed to expanding the dataset for training the surrogate model. Given that the element size in the coarser model M5 was four times larger than that in M1, the computational cost was reduced by a factor $4^{2}$. However, this efficiency gain came at the expense of accuracy—errors in M5 increased by factors of $4^{4}$. While this loss of precision was substantial, the proposed methodology was designed to account for and mitigate this issue.

The pseudo-experimental model was derived from the M1 FE model, where the computed natural frequencies underwent the following nonlinear transformation:

(8) $$f^{\mathrm{Me}}=f^{M1}+20\cdot \sin\left(\frac{1}{60}\cdot f^{M1}-5\right)=\mathrm{Me}\left(f^{M1}\right),$$

where $f^{M1}$ represents the vector of natural frequencies (in Hz) obtained from the M1 model, corresponding to specific mode shapes $\phi_{i}$ within the mode shape matrix Φ (see [40]). Unlike a typical approach where the frequency vector contains the lowest natural frequencies in sequential order, in this study, it included only frequencies corresponding to selected mode shapes. To enable such selection, the mode shapes obtained from numerical simulations first had to be identified and subsequently filtered to retain only the eleven most relevant ones [40].

This strategy enhanced the accuracy of the surrogate model by focusing on the most meaningful vibrational modes and eliminating unnecessary information that could introduce noise into the learning process. As a result, the optimization procedure benefited from improved convergence and solution quality, as demonstrated in the authors’ previous studies [40].

The transformed vector $f^{\mathrm{Me}}$ represents the pseudo-experimental model’s natural frequencies, and the function Me(·) mimics experimental testing procedures. The neural network-based approximation—surrogate model application—of $f^{\mathrm{Me}}$ is denoted as $f_{\mathrm{SM}}^{\mathrm{Me}}$.

It is important to note that the function Me(·) does not stem from actual experimental research but is instead an attempt to model discrepancies between numerical simulations and laboratory experiments. The authors’ previous studies relied entirely on numerical analyses; thus, incorporating the pseudo-experimental model into the optimization framework enables the consideration of potential deviations encountered in experimental studies. Furthermore, this approach helps address practical limitations related to the number of feasible experimental tests.

### 2.5. Optimization Strategy Using Genetic Algorithms, Surrogate Models, and Curriculum Learning

The optimization problems given by Equation (6) were herein solved using the Non-dominated Sorting Genetic Algorithm II (NSGAII) [41], a GA-based multi-objective search method that is not derivative-based. Genetic algorithms are widely used in complex engineering problems, particularly where traditional optimization methods prove insufficient [6,40]. They work on a population of possible solutions and use deterministic computations and random number generators. The GA’s advantage, crucial from the point of view of the problem to be solved, is the ability to search the entire solution space when trying to find a global minimum. However, this requires repeated evaluations of the objective function, which is computationally expensive when the FEM is applied. In the proposed optimization procedure, the objective function was solved using a surrogate model instead of FEM calculations. Therefore, the GA procedure worked extremely fast.

However, one of the key challenges associated with GAs is the need to repeatedly evaluate the objective functions. This process can be computationally expensive, especially when the objective functions require time-consuming numerical analyses, such as FEM simulations. To significantly mitigate this issue, the present approach employed surrogate models based on deep neural networks (DNNs).

The use of DNNs as surrogate models enables the rapid approximation of analysis results, replacing costly simulations with near-instantaneous predictions. This allows for large-scale optimization while drastically reducing computation time. The effectiveness of this approach was confirmed in the authors’ previous studies, where it was demonstrated that using a DNN for objective function prediction led to a significant reduction in computational burden compared to conventional methods [42].

The process of selecting DNN parameters required a thorough evaluation of network errors, taking into account the following aspects:

- The number of input variables, denoted as *I*;
- The number of layers, represented by $N_{L}$;
- The number of neurons *H* within each hidden layer (expressed as $H^{\left(\cdot \right)}$, maintaining consistency across all hidden layers within a specific network);
- The number of output nodes, denoted as *O*;
- The choice of learning algorithms and regularization techniques, along with other contributing factors;
- The choice of activation and loss functions.

A summary of the different network parameter values considered is presented in Table 2. It is worth noting that the architecture 17-50-50-50-11, in combination with the Tanh activation function and the RMSProp learning algorithm, provided optimal performance in over 80% of the evaluated DNNs. This configuration was frequently used alongside Batch Normalization (BN) for regularization and Early Stopping strategies. Also, the best results were achieved using the MAE as the loss function.

Preparing surrogate models in the form of a DNN requires generating an appropriate dataset for training. To achieve this, an MF approach was introduced to limit the number of calls to the high-fidelity M1 model during data generation. The less accurate M5 model was employed, allowing the acquisition of a large number of training samples at the cost of reduced accuracy. In the authors’ previous study [43], it was demonstrated that increasing the FE size by a factor of *h* resulted in an approximately $h^{2}$ reduction in computational time. However, this simplification came at the expense of accuracy, as the numerical error increased by a factor of $h^{4}$. This trade-off underscores the necessity of incorporating correction mechanisms, such as auxiliary neural networks, to mitigate the errors introduced by the lower-fidelity M5 model. The number of cases computed using the M1 model (which also provided pseudo-experimental data samples) was an order of magnitude smaller than the number of cases evaluated with the M5 model. To further enhance the accuracy of the surrogate model, auxiliary neural networks were incorporated to compensate for the errors introduced by the lower-fidelity M5 model. The number of M1 model evaluations was denoted as $n_{M1}$, while the number of M5 model evaluations was denoted as $n_{M5}$.

**Table 2:** Architecture, algorithms, function, and methods used in DNN simulations [44].

DNN architecture	I={17,28}
$I-H^{\left(\cdot \right)}-O$	$N_{L}=\left\{4,5,6,7,8\right\}$
	$H^{\left(\cdot \right)}=\left\{20,30,40,50,75,100\right\}$
	O=11
Learning algorithms	ADAM
	* RMSProp
	SGD
Regularization methods	* Early Stopping
	$L_{2}$ Regularization
	Dropout
	* Batch Normalization
Activation functions	SoftMax
	* Tanh
	ReLu
	Sigmoid
Loss functions	MSE
	* MAE
	ArcSin

* Option selected based on preliminary testing.

Within this framework, two FEM models of varying accuracy were utilized: a high-fidelity model (M1) and a low-fidelity model (M5). The M1 model served as a reference and was used to generate pseudo-experimental data by introducing a nonlinear perturbation function Me(·). This function aimed to account for potential discrepancies between numerical results and real experimental data, thereby enabling optimization under conditions closer to real-world scenarios. This introduced an additional verification step, allowing for the assessment of the robustness of the applied optimization methods against inevitable errors and uncertainties present in experimental data. The low-fidelity model M5, on the other hand, facilitated the rapid estimation of preliminary values while significantly reducing computational costs.

The integration of MF modeling with deep neural networks enhanced the efficiency of the surrogate model training process, allowing for more precise representation of dependencies in the design space, while maintaining an acceptable computation time. The following sections provide a detailed discussion of three different approaches, each varying in the construction of surrogate models and their integration with the optimization procedure.

**Figure 2:**
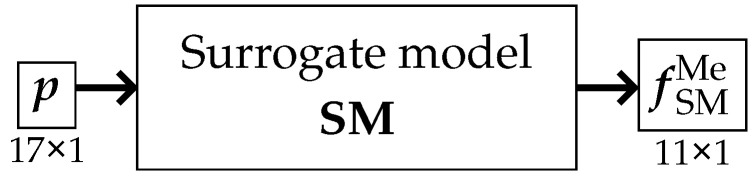
The surrogate model.

Regardless of the applied variant, the primary objective of the surrogate model remained unchanged. Its purpose was to predict the pseudo-experimental frequency values $f^{\mathrm{Me}}$ based on a given vector of model parameters p. Ultimately, regardless of the methodology adopted for constructing and training the surrogate model, its operation can be symbolically depicted as in Figure 2.

**Figure 3:**
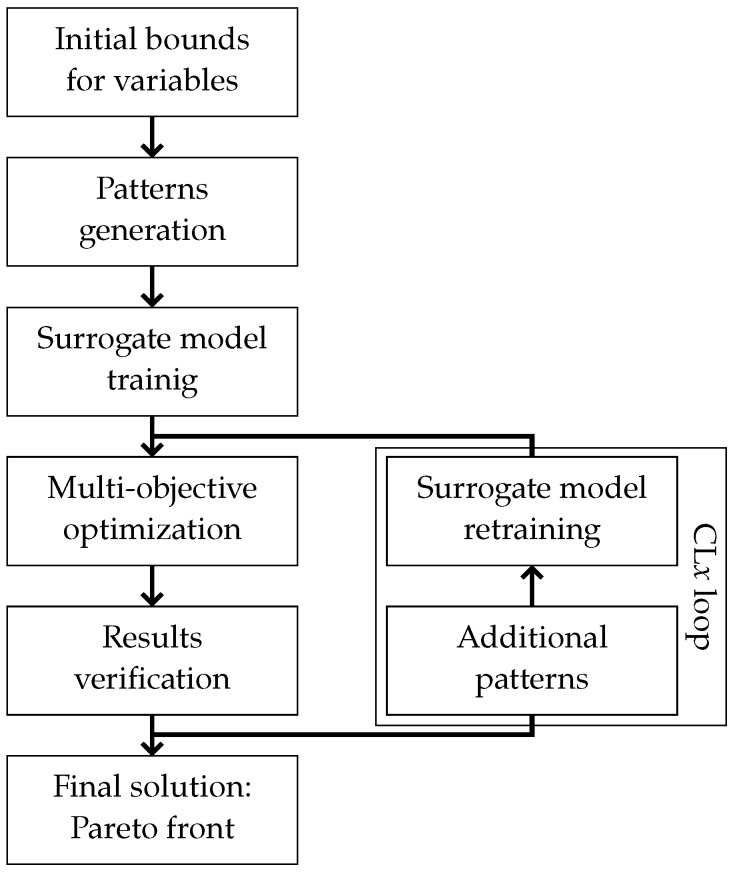
The optimization scheme, including CL*x* loops.

The optimization procedure, whose concept is presented in Figure 3, was based on an iterative approach involving multiple refinements of the surrogate model within the framework of CL.

The process begins with data generation, which includes a large number of samples obtained using the simplified M5 model ($n_{M5}$) and a significantly smaller number of samples derived from the pseudo-experimental Me(M1) model ($n_{M1}$). This approach allows for the collection of a comprehensive dataset while simultaneously limiting the computational cost associated with the high-fidelity M1 model.

Based on the generated dataset, a surrogate model in the form of a deep neural network is constructed and appropriately trained. Once the training process is completed and the surrogate model is prepared, the optimization procedure is initiated using a GA. At that stage, the surrogate model plays a crucial role in enabling the efficient and rapid estimation of the objective function values.

After completing the first optimization cycle, the obtained results are validated and subsequently used to build an additional dataset. The new samples focus on regions of the design space located in the vicinity of the optimal solution, facilitating the further refinement of the surrogate model.

In the subsequent steps, the surrogate model is retrained based on the newly generated data, and the optimization process is restarted, this time utilizing the improved surrogate model. The iterative refinement cycles of the surrogate model form the core of the CL approach, where *x* represents the number of performed iterations.

The procedure terminates after reaching a predefined number of CL cycles, ensuring a systematic improvement in the quality of the surrogate model and yielding the final optimized solution.

#### 2.5.1. Variant I

**Figure 4:**
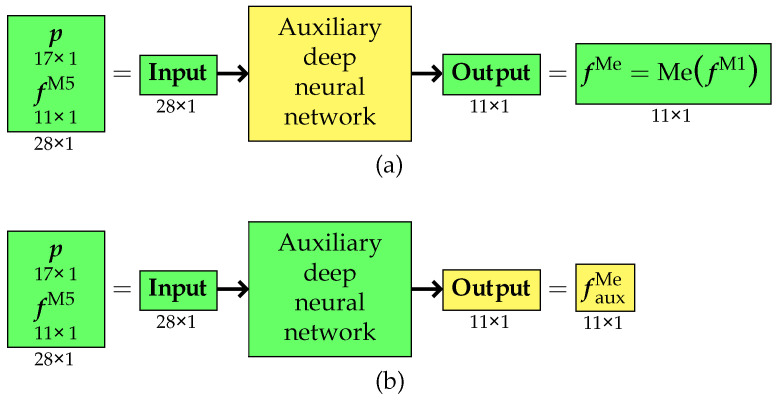
Variant I: (**a**) training and (**b**) application phase of the auxiliary surrogate model.

In the first approach variant, an auxiliary surrogate model was first developed to generate training data for the primary surrogate model. The purpose of the auxiliary model was to refine the results obtained from the low-fidelity M5 model so that they would closely match the values derived from the pseudo-experimental model $\mathrm{Me}\left(f^{M1}\right)$. To achieve this, FEM calculations were performed for a limited number of cases using both the high-fidelity M1 model and the low-fidelity M5 model. Based on the collected data, an auxiliary model was trained. Its inputs consisted of the structural design parameters, gathered in the vector p, along with a vector $f^{M5}$ of eleven selected natural frequencies obtained from the M5 model. The neural network was trained to accurately estimate the pseudo-experimental frequencies $f^{\mathrm{Me}}=\mathrm{Me}\left(f^{M1}\right)$, which served as approximations of real experimental measurements (see Figure 4a).

Upon completion of the training process, the trained auxiliary surrogate model was used to predict pseudo-experimental frequency values $f_{\mathrm{aux}}^{\mathrm{Me}}$ based on the results from rapid calculations using the M5 model only (see Figure 4b).

**Figure 5:**
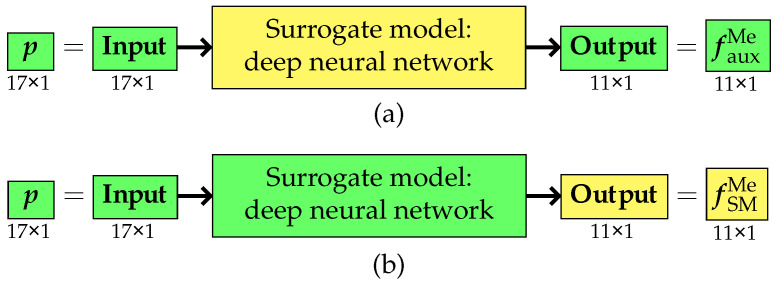
Variant I: (**a**) training and (**b**) application phase of the primary surrogate model.

This approach enabled the generation of a large dataset, which was subsequently used to train the primary surrogate model (see Figure 5a). The role of this final surrogate model was to predict pseudo-experimental frequency values $f_{\mathrm{SM}}^{\mathrm{Me}}$ solely based on the design parameter vector p, eliminating the need for any additional numerical simulations (see Figure 5b).

This methodology significantly reduced the necessity of repeatedly utilizing the computationally expensive M1 model (as well as the pseudo-experimental model). Moreover, it facilitated the development of an accurate and efficient primary surrogate model. The large number of training samples generated by the auxiliary model allowed for precise predictions while maintaining a low computational cost.

#### 2.5.2. Variant II

In the second approach, a different architecture was employed for the auxiliary surrogate model, while the primary surrogate model remained unchanged from the first variant. The key modification introduced in this version was the division of the auxiliary neural network structure into two distinct modules: one dedicated exclusively to processing linear dependencies and the other responsible for capturing nonlinear components of the mapping. Despite its more complex architecture, the auxiliary surrogate model remained a single neural network.

This architectural choice for the auxiliary model was based on the assumption that for functions that can be decomposed into linear and nonlinear components, processing these elements separately should yield more accurate approximation results [45,46,47]. By structuring the auxiliary model in this manner, it was possible to better align its design with the characteristics of the data, thereby improving its ability to capture the relationships between structural parameters and the resulting pseudo-experimental frequencies.

**Figure 6:**
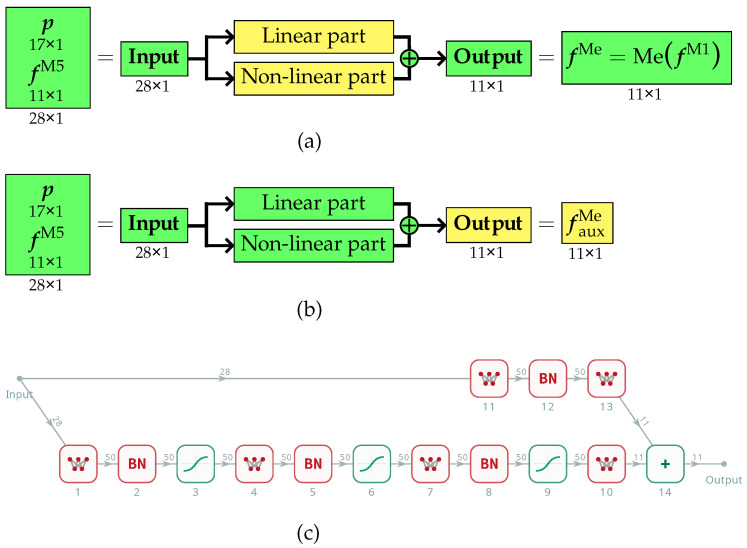
Variant II: (**a**) training and (**b**) application phase of the auxiliary surrogate model; (**c**) the structure of the applied two-module DNN.

The training procedure of the auxiliary surrogate model (see Figure 6a), its application phase (see Figure 6b), and its objective remained identical to those in the first variant. The precomputed values from the simplified M5 model were still utilized and subsequently corrected using the trained network to best match the values obtained from the pseudo-experimental model. The refined data were then used to construct the main surrogate model, whose purpose was to estimate the pseudo-experimental frequency values $f_{\mathrm{SM}}^{\mathrm{Me}}$ based solely on the design parameter vector p, eliminating the need for multiple costly numerical computations (see Figure 5b).

A similar modular architecture to the one described above for the auxiliary surrogate model (see Figure 6c) was also tested for the primary surrogate model. The goal was to examine whether separating linear and nonlinear processing could enhance the accuracy of pseudo-experimental frequency predictions. However, the results obtained with this configuration did not show significant improvements over the standard approach, and in some cases, even led to increased approximation errors in the surrogate model. Consequently, this approach was abandoned.

#### 2.5.3. Variant III

The third variant of the approach differed significantly from the two previous methods. It still utilized two surrogate models; however, their role and application underwent substantial modifications. Unlike variants I and II, where the auxiliary surrogate model was used solely for preparing training data for the primary surrogate model, in this approach, both models were employed simultaneously and actively participated in the entire optimization process.

The first surrogate model was designed to replace computations performed using the simplified M5 model. Its primary function was to directly estimate the selected natural frequencies $f^{M5}$ obtained originally from the M5 model based on the vector of design parameters p. This eliminated the need for the repeated use of the M5 model during the optimization process.

**Figure 7:**
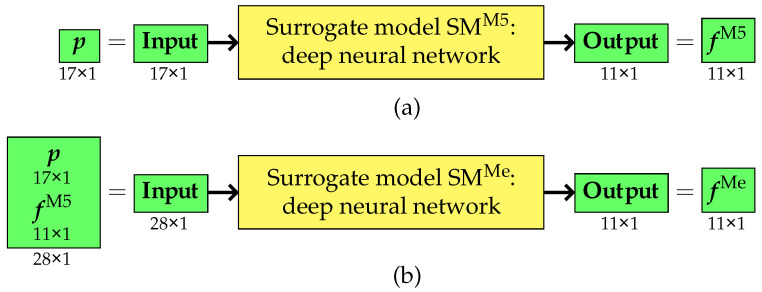
Variant III: training phase of (**a**) the first surrogate model and (**b**) the second surrogate model.

**Figure 8:**
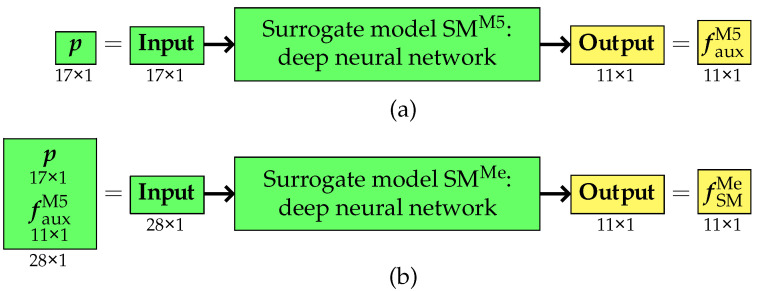
Variant III: application phase of (**a**) the first surrogate model and (**b**) the second surrogate model.

The second surrogate model, in turn, was responsible for estimating the pseudo-experimental frequencies $f^{\mathrm{Me}}$, which are essential for optimization. Its input consisted of an extended input vector comprising both the design parameter vector p and the vector of frequencies $f_{\mathrm{aux}}^{M5}$ obtained from the first surrogate model. As a result, this model accounted for both the structural characteristics and the dynamic properties derived from the analysis of the M5 model (or, more precisely, from the first surrogate model). The training and application of both surrogate models is presented in Figure 7 and Figure 8.

With this configuration, both surrogate models were utilized at every stage of the optimization process.

### 2.6. Indicators: Pareto Front Quality Metrics

The results of the multi-objective optimization problem analyzed in this study, which involved two objective functions, resulted in a two-dimensional Pareto front.

For an objective assessment of the quality of solutions obtained through multi-objective optimization, appropriate evaluation metrics must be introduced. While visual inspection of several Pareto fronts is effective for distinguishing qualitative differences, it becomes insufficient when variations between the compared fronts are merely quantitative. In such cases, the repeated comparison of Pareto fronts necessitates the definition of numerical quality metrics. These indicators allow for an objective evaluation of various characteristics of the analyzed fronts. Audet et al. [30] reviewed a total of 57 performance indicators and categorized them based on the evaluated parameters into four groups: (i) cardinality indicators, (ii) convergence indicators, (iii) distribution and spread indicators, and (iv) convergence and distribution indicators. Alternatively, Tian et al. [48] proposed a more simplified classification, distinguishing only between (i) diversity indicators (assessing the evenness and spread of the Pareto front) and (ii) convergence indicators.

In this study, four indicators were selected. The first was the hypervolume indicator, denoted as $I_{H}$, and the second was the relative hypervolume indicator, denoted as $I_{H}^{r}$. The hypervolume indicators are classified as convergence and distribution indicators in [30] or as convergence and diversity indicators in [48]. The hypervolume indicator $I_{H}$ is recognized as the most widely used metric [29]. The third metric utilized was the Epsilon ϵ-indicator [49], referred to as $I_{\epsilon }$. It is classified as a convergence indicator in [30] and ranks as the third most frequently used indicator according to [29]. The second most common metric, the Generational Distance indicator, was applied in this study as the fourth indicator.

Originally introduced by Zitzler [50], the hypervolume indicator measures the area covered by the examined Pareto front *A* relative to a suitably chosen reference point. When comparing two fronts, *A* and *B*, this indicator can be adapted as the difference $I_{H}\left(A\right)-I_{H}\left(B\right)$. If one of the compared fronts represents the true Pareto front (TPF), meaning the optimal front sought during the optimization process, the indicator can be redefined as a unary metric: $I_{H2}\left(A\right)=I_{H}\left(TPF\right)-I_{H}\left(A\right)$. The relative hypervolume indicator used in this study is given by:

(9) $$I_{H}^{r}=\frac{I_{H}\left(TPF\right)-I_{H}\left(A\right)}{I_{H}\left(TPF\right)},$$

where $I_{H}\left(TPF\right)$ and $I_{H}\left(A\right)$ denote the areas covered by the TPF and the examined Pareto front *A*, respectively. The true Pareto front was defined in this study as the envelope of the results obtained from all examined approaches and variants considered in the analysis. Therefore, it did not represent a fully legitimate TPF, which should ideally be derived analytically. Instead, it served as the most accurate possible approximation of the true optimal front within the scope of this study.

The third selected indicator, $I_{\epsilon }\left(A,B\right)$, represents the smallest scalar ϵ that scales Pareto front *B* so that every point in ϵ·B is dominated by at least one point in *A*. If the second Pareto front corresponds to the TPF, this metric can be treated as a unary indicator, denoted as $I_{\epsilon 1}\left(A\right)$, which was applied in this form in the present study.

The fourth selected indicator, the Generational Distance indicator $I_{GD}$ [51] measures the average distance of the obtained Pareto front solutions from the TPF and is defined as:

(10) $$I_{GD}={\left(\frac{1}{N}\sum_{i=1}^{N}d_{i}^{2}\right)}^{\frac{1}{2}}$$

where

- *N* is the number of points in the approximated Pareto front;
- $d_{i}$ is the distance of each solution from the nearest point in the TPF.

## 3. Results

### 3.1. Evaluation of High-Fidelity Sample Size and Training Strategies for Surrogate Models

**Figure 9:**
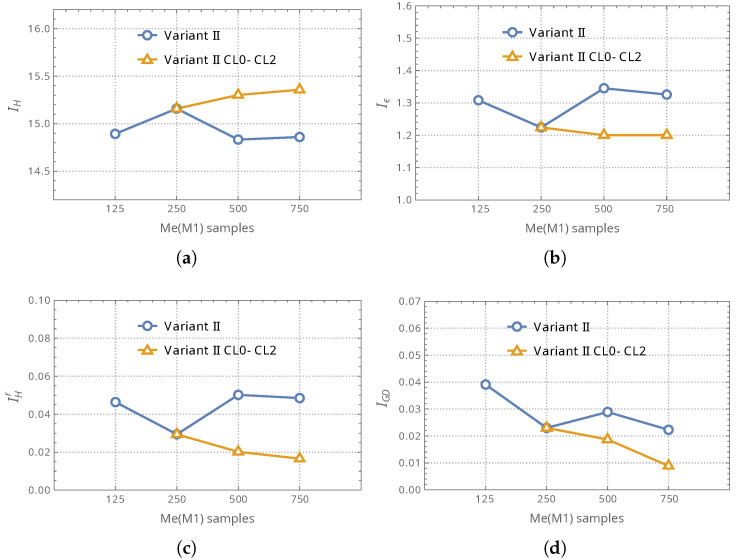
The optimization outcomes obtained using a surrogate model trained with a single-step training (no CLx) or CL iterative approach: (**a**) $I_{H}$, (**b**) $I_{\epsilon }$, (**c**) $I_{H}^{r}$, (**d**) $I_{GD}$.

The initial analyses aimed to determine the optimal number of M1 samples in the training dataset for the surrogate model. Table 3 and Figure 9 present the first set of results, where the table provides numerical values, and the figures offer a graphical representation with the vertical axes representing the values of four selected indicators and the horizontal axes indicating the number of M1 samples used in the applied datasets. For the reader’s convenience, the desired trend for each indicator is repeated in parentheses (higher or lower values preferred):

- Hipervolume indicator $I_{H}$: see Figure 9a (the higher the better),
- ϵ indicator $I_{\epsilon }$: see Figure 9b (the lower the better),
- relative Hipervolume indicator $I_{H}^{r}$: see Figure 9c (the lower the better),
- Generational Distance indicator $I_{GD}$: see Figure 9d (the lower the better).

The analysis of these indicators enabled the evaluation of the effectiveness of successive iterations in improving the surrogate model and their impact on the final quality of the optimization outcomes.

This analysis focused on comparing the optimization outcomes obtained using a surrogate model trained with a nearly identical number of computationally expensive Me(M1) samples but employing two distinct training strategies: (i) a single-step training approach utilizing all randomly generated samples at once, and (ii) an iterative refinement approach based on CL.

The tests presented in the table and figures were conducted exclusively for the second variant of the surrogate model, in which the auxiliary neural network consisted of separate modules dedicated to processing linear and nonlinear dependencies. It is important to note that Figure 9 does not encompass the entire range of simulations performed within the CL procedure. The results displayed in the figures correspond only to those cases for which calculations were also performed using a surrogate model trained without CL iterations, allowing for a direct comparison.

**Figure 10:**
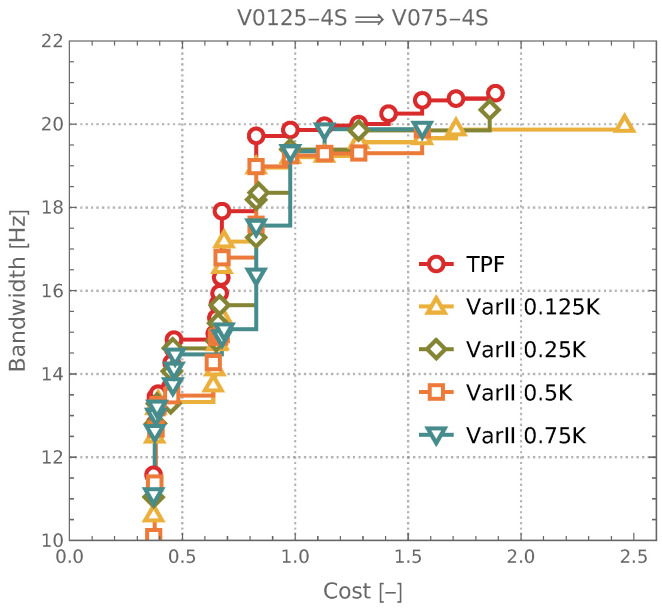
Comparison of Pareto fronts obtained without CL iterations, using different numbers of high-quality samples for surrogate model training.

The results obtained for the variant that did not utilize CL are additionally presented not only in the form of Pareto front quality indicators but also through the resulting Pareto fronts themselves. A graphical representation of these fronts is provided in Figure 10, allowing for a direct comparison of their shape and distribution.

The analysis of the results shown in Figure 9a–d leads to several key conclusions. First and foremost, there is no need to use more than 250 high-fidelity M1 samples in the initial stage of optimization (CL0), as increasing their number did not improve results and could even degrade performance in some cases. Comparing Pareto fronts obtained from different approaches that did not incorporate CLx loops (see Figure 10) was more complex; however, even in this case, the benefits of using 250 M1 samples could be observed. Another important finding is the impact of successive CLx loops on optimization quality. The conducted analyses demonstrated that the iterative refinement of the surrogate model led to a noticeable improvement in all applied performance indicators, as confirmed for CL1 and CL2 iterations. These findings highlight the effectiveness of the proposed approach and confirm that the key factors influencing the quality of the surrogate model are the proper management of high-fidelity samples and the application of iterative learning.

### 3.2. Evaluation of Different Surrogate Model Configurations at CL0 Stage

**Figure 11:**
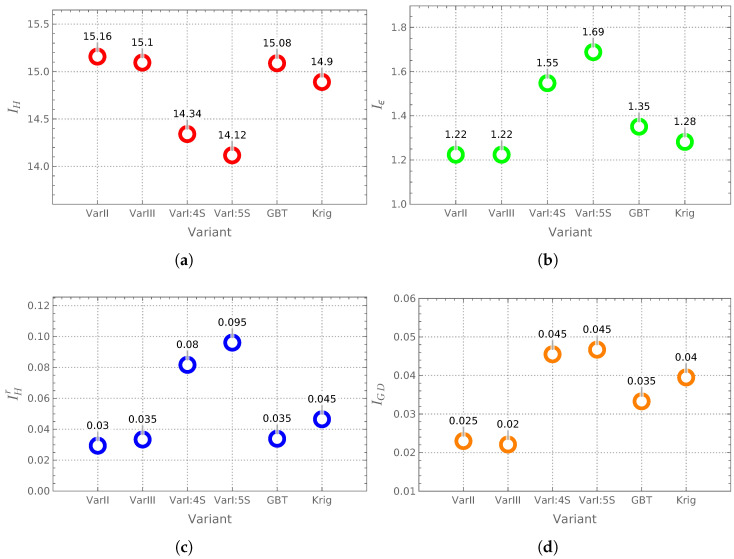
Comparison of performance indicators for different surrogate model variants at the CL0 stage: (**a**) $I_{H}$, (**b**) $I_{\epsilon }$, (**c**) $I_{H}^{r}$, (**d**) $I_{GD}$.

In the next step, a preliminary comparison (see Figure 11) was conducted at the CL0 stage (i.e., without refinement loops improving the model’s accuracy) to evaluate the results obtained from three different surrogate model construction approaches (Variants I through III). Additionally, two alternative surrogate models were examined, where the auxiliary neural network (see Variant I, Figure 4a) was replaced by either Gradient Boosted Trees (GBTs) or Kriging inference (Krig). In this comparison, 250 high-fidelity M1 samples and 4000 low-fidelity, corrected M5 samples, were used. The only exception was the case labeled as “VarI: 5S”, where the number of M5 samples was increased to 5000.

The analysis of the obtained results indicates that the best optimization outcomes were achieved using Variant II and Variant III surrogate models.

A comparison of the results obtained for the Variant I surrogate model (denoted in the figures as “VarI: 4S” and “VarI: 5S”) suggests that employing 4000 M5 samples was justified. A further analysis of these cases demonstrated that increasing the number of M5 samples to 5000 (as in “VarI: 5S”) did not provide significant improvements in result quality.

Additionally, the results obtained using Gradient Boosted Trees (GBTs) and Kriging (Krig) indicated that in the Variant I surrogate model, during the construction phase of the auxiliary surrogate model (Figure 4a), alternative machine learning methods could be applied. This was feasible because the number of training samples in this phase was relatively small (250), allowing the effective use of techniques such as GBTs and Kriging. However, in the second phase (where the number of training samples increased to 4000), the results obtained using GBTs and Krig significantly deteriorated and were unsuitable for optimization purposes. Consequently, a DNN (deep neural network) was selected as the final surrogate model.

This finding suggests the potential for a hybrid approach, where GBTs or Kriging could be applied in the initial phase, followed by a DNN as the final surrogate model (GBT→DNN or Krig→DNN). However, this hybrid methodology was not further explored in the present study.

### 3.3. Analysis of Effectiveness of Optimization Utilizing Curriculum Learning

**Figure 12:**
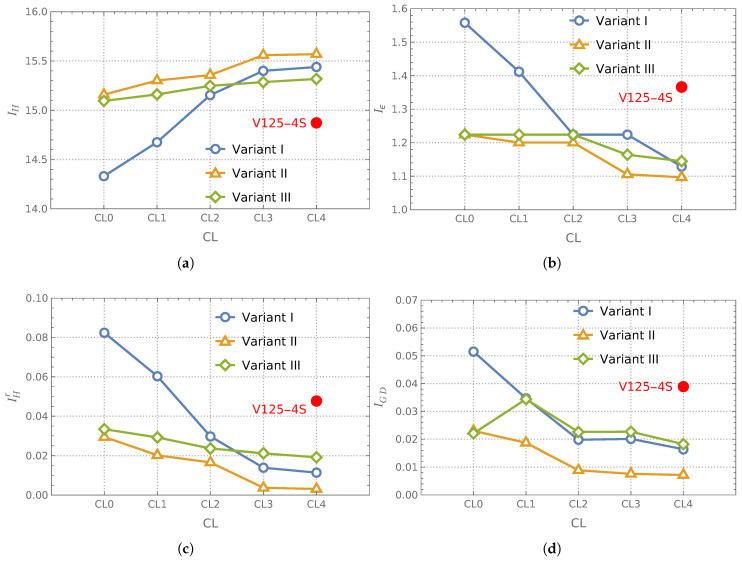
Surrogate model variants and Pareto front indicators obtained for subsequent CL loops: (**a**) $I_{H}$, (**b**) $I_{\epsilon }$, (**c**) $I_{H}^{r}$, (**d**) $I_{GD}$.

This section presents the results of the analysis of three surrogate model variants, each subjected to an iterative refinement process within the framework of CL; see Figure 12. The plots in the subsequent subfigures show the values of the applied performance indicators (vertical axes) as a function of the number of applied CL loops (horizontal axes). A detailed analysis was conducted for the case where the number of M1 samples (and consequently Me samples) was 250, while the number of M5 samples was 1000. This configuration is symbolically referred to as V025-4S. In each case of different surrogate models variants, five optimization cycles were conducted, denoted as CL0, CL1, CL2, CL3, and CL4. The results are presented in figures summarizing the tendencies of four selected performance indicators.

Each CL iteration required verification of results, allowing for the preparation of a new batch of training samples. In the applied approach, each CL iteration introduced approximately 250 additional training samples. Consequently, iterations from CL0 to CL3 resulted in around 1000 new samples generated using the M1 model, which was utilized for verification.

The surrogate models applied in the CL0 iteration were trained on an initial dataset containing 250 Me(M1) samples. After completing the CL3 stage, these models were further refined through four additional training phases, each incorporating approximately 250 new samples. With reasonable approximation, it can be stated that the surrogate models used in the CL4 iteration were trained on a dataset containing a total of 250+4×250=1250 samples of M1 quality.

Additionally, this study examined an alternative approach in which a surrogate model, built according to Variant I, was trained using all 1250 M1 simulation samples from the outset, without iterative refinement through CL cycles. In Figure 12, the results of this approach are marked with a red dot labeled V125-4S (1250 high-quality M1 samples and 4000 lower-quality auxiliary network-refined M5 samples). Due to the comparable number of high-fidelity samples, these results are presented in the CL4 column. However, it should be noted that formally, these correspond to CL0 since no iterative learning process was applied. Nevertheless, the number of high-fidelity samples used in this approach closely matched that of the V025-4S configuration at the CL4 stage.

For the reader’s convenience, the numerical results presented graphically in Figure 12 are additionally provided in Table 4. To clarify the interpretation of the data presented in the table, several explanations are necessary. The columns labeled CL1, CL2, CL3, and CL4 display the values obtained in successive iterations of the CL process. The values in parentheses within these columns indicate the improvement achieved compared to the previous step, allowing for an assessment of the effectiveness of each iterative refinement in the surrogate model training process. The second-to-last column represents the overall improvement between CL4 and CL0. Additionally, the last column presents the overall improvement in results obtained using Variant I or Variant II relative to Variant III. The values in this column provide insight into which surrogate model approach yielded superior final results and quantify the extent to which Variant I or Variant II outperformed Variant III.

**Figure 13:**
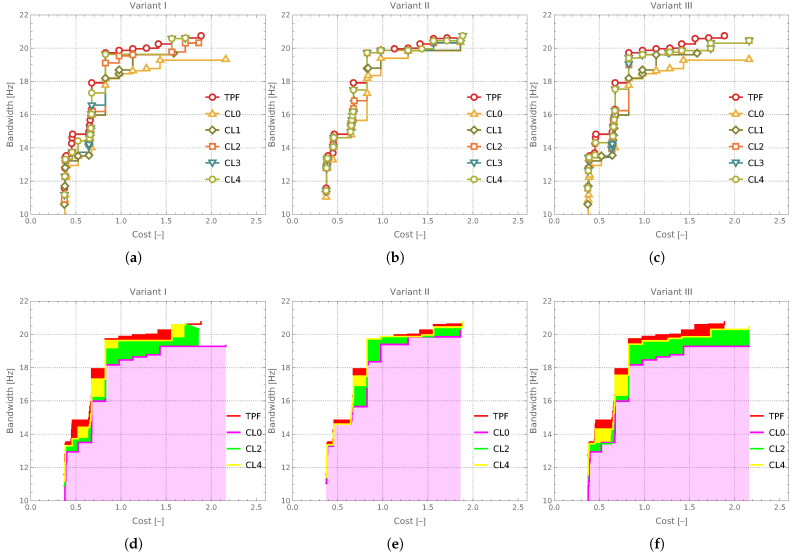
Pareto fronts obtained for subsequent CL loops: (**a**,**d**) Variant I, (**b**,**e**) Variant II, (**c**,**f**) Variant III.

The Pareto fronts (not their indicators, as previously) obtained from the optimization process using different surrogate model variants and varying numbers of CL loops are presented in Figure 13a–c. These plots also include the TPF, which serves as a reference (benchmark) for assessing the quality of the obtained solutions.

An alternative visualization of the same results (displayed only for every second CL loop) is provided in Figure 13d–f. The magenta color is used to indicate the region enclosed by the Pareto front obtained for CL0. The green-shaded region corresponds to the area bounded by the Pareto front obtained in the CL2 iteration. However, only those portions of this region where the CL2 front dominated over the CL0 front are visible in the figure. Notably, the front obtained in CL2 was never inferior to the one from CL0. Similarly, regions where the Pareto front from CL4 outperformed the front from CL2 are highlighted in yellow. Finally, the red color marks areas where the True Pareto Front (TPF) provided superior results compared to the front obtained in CL4.

**Figure 14:**
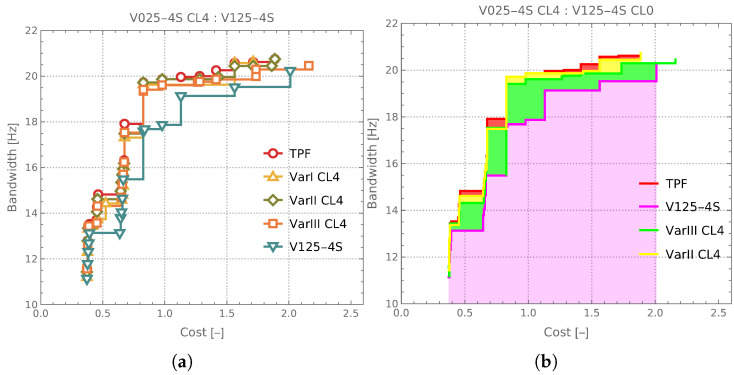
Pareto fronts obtained for CL4 loops and CL0 case V125-4S: (**a**) line-based representation of Pareto fronts, (**b**) surface-based representation of Pareto fronts.

The next figure, namely, Figure 14, presents the Pareto fronts obtained after the CL4 iteration for each of the considered surrogate model variants, as well as the Pareto front corresponding to the V125-4S case, previously described in the context of Figure 12. In the four cases depicted in the figure (supplemented by the TPF), the number of Me samples, which required computationally expensive evaluations, was very similar, amounting to approximately 1250.

This figure provides a clear assessment of the quality of the CL0 approach (where no CL iterations were applied, as in the case of V125-4S) compared to the iterative improvement strategy, in which the surrogate model was refined through successive CL loops.

## 4. Discussion

The analytical mode shape identification procedure employed in this study did not achieve the same level of precision for the analyzed geometries as it did for the originally considered cylindrical structure, on which it was initially developed (see [40,52]). This method involved identifying the node with the highest displacement for a given mode shape and determining the corresponding vibration mode based on the displacement direction and comparison with selected reference points. The application of concave or convex hyperboloid geometries introduced additional challenges in the identification process, as the curvature variation affected the displacement patterns and complicated the interpretation of mode shapes.

Although the identification accuracy could potentially be improved by fine-tuning selected parameters and coefficients within the identification procedure, the authors recognized this as an opportunity to assess the robustness of the proposed optimization framework in the presence of identification errors. While for a cylindrical structure, the identification method proved highly effective, with an estimated error rate of only about 1%, the more complex hyperboloid of revolution, which could exhibit both concave and convex configurations, resulted in a significantly higher error rate, increasing by several times.

Despite this increased error rate, the optimization process remained stable and effective, demonstrating its resilience to imperfections in mode shape identification. The observed identification inaccuracies did not introduce critical disruptions in the algorithm’s performance, further validating the applicability of the proposed approach to optimizing complex geometries.

## 5. Conclusions

Based on the presented results, the following key conclusions can be drawn:

1. Effectiveness of CL iterations: The introduction of CL loops significantly enhanced the optimization outcomes. The results demonstrated that iterative refinement of the surrogate model through CL1 and CL4 led to a noticeable improvement in all applied performance indicators. This confirmed the effectiveness of CL in refining surrogate models and improving optimization performance.
2. Pareto front analysis: The visualization of Pareto fronts obtained for different surrogate models and CL iterations confirmed the positive impact of iterative learning on optimization quality. Moreover, the comparison of Pareto fronts from CL4 iterations with the V125-4S approach provided insights into the advantages of an iterative model refinement strategy over direct surrogate model training with a large dataset.
3. Comparison of different surrogate model variants: The best optimization results were achieved using the surrogate models from Variant II, then Variant I. These models consistently outperformed Variant III in terms of Pareto front quality indicators. Additionally, the comparison of Variant I results confirmed that increasing the number of low-fidelity samples above 4000 did not yield significant benefits.
4. Optimal number of high-fidelity samples: The analyses indicated that using more than 250 high-fidelity M1 samples in the initial optimization stage (CL0) did not improve results and, in some cases, could even degrade performance. This suggests that the selection of an appropriate number of high-fidelity samples is crucial for balancing computational cost and optimization accuracy.
5. Evaluation of alternative machine learning approaches: the findings suggest that, in the auxiliary surrogate model construction phase, alternative machine learning methods such as GBTs and Kriging can be effectively used.

These findings underscore the importance of iterative refinement in surrogate-based optimization and suggest that a carefully structured training approach, incorporating CL, can significantly enhance optimization performance while maintaining computational efficiency.

## Figures and Tables

**Table 1:** The properties of three fiber-reinforced composite materials: CFRP, GFRP and *t*FRP (see [38]).

Material	μ	$E_{a}$	$E_{b}$	$E_{c}$	$\nu_{\mathrm{ab}}$	$\nu_{\mathrm{ac}}$	$\nu_{\mathrm{bc}}$	$G_{\mathrm{ab}}$	$G_{\mathrm{ac}}$	$G_{\mathrm{bc}}$	Mass Density	Cost
Label	[-]	[GPa]	[GPa]	[GPa]	[-]	[-]	[-]	[GPa]	[GPa]	[GPa]	[$\mathrm{kg}/m^{3}$]	[-]
CFRP	1	120	8	8	0.014	0.028	0.028	5	5	3	1536	10.20
*t*FRP	2	80	6	6	0.020	0.036	0.036	4	4	3	1428	5.78
GFRP	3	40	4	4	0.026	0.044	0.028	3	3	3	1320	1.36

**Table 3:** The optimization outcomes obtained using a surrogate model trained with a single-step training Variant II (no CLx) or CL iterative approach on Variant II CL0–CL2.

		Me(M1) Samples			
		125	250	500	750
$I_{H}$	Variant II	14.8924	15.1581	14.8327	14.8594
	Variant II CL0–CL2	—	15.1581	15.3016	15.3572
“Variant II CL0–CL2” improvement vs. “Variant II”				3%	3%
$I_{\epsilon }$	Variant II	1.3082	1.2241	1.3453	1.3258
	Variant II CL0–CL2	—	1.2241	1.2004	1.2004
“Variant II CL0–CL2” improvement vs. “Variant II”				11%	9%
$I_{H}^{r}$	Variant II	0.0464	0.0294	0.0502	0.0485
	Variant II CL0–CL2	—	0.0294	0.0202	0.0166
“Variant II CL0–CL2” improvement vs. “Variant II”				60%	66%
$I_{GD}$	Variant II	0.0391	0.0230	0.0289	0.0223
	Variant II CL0–CL2	—	0.0230	0.0187	0.0089
“Variant II CL0–CL2” improvement vs. “Variant II”				35%	60%

**Table 4:** Surrogate model variants and Pareto front indicators obtained for subsequent CL loops.

							CL4	Improvement
		CL0	CL1	CL2	CL3	CL4	vs. CL0	vs. Variant III
	Variant I	14.331	14.676 (2%)	15.153 (3%)	15.400 (2%)	15.439 (0%)	8%	1%
$I_{H}$	Variant II	15.158	15.302 (1%)	15.357 (0%)	15.559 (1%)	15.569 (0%)	3%	2%
	Variant III	15.095	15.161 (0%)	15.248 (1%)	15.286 (0%)	15.318 (0%)	1%	—
	Variant I	1.5580	1.4119 (9%)	1.2241 (13%)	1.2241 (0%)	1.1288 (8%)	28%	1%
$I_{\epsilon }$	Variant II	1.2241	1.2004 (2%)	1.2004 (0%)	1.1057 (8%)	1.0968 (1%)	10%	4%
	Variant III	1.2241	1.2241 (0%)	1.2241 (0%)	1.1640 (5%)	1.1449 (2%)	6%	—
	Variant I	0.0824	0.0603 (27%)	0.0297 (51%)	0.0139 (53%)	0.0114 (18%)	86%	41%
$I_{H}^{r}$	Variant II	0.0294	0.0202 (31%)	0.0166 (18%)	0.0037 (78%)	0.0031 (16%)	89%	84%
	Variant III	0.0334	0.0292 (13%)	0.0236 (19%)	0.0212 (10%)	0.0192 (9%)	43%	—
	Variant I	0.0515	0.0347 (33%)	0.0198 (43%)	0.0201 (−2%)	0.0164 (18%)	68%	10%
$I_{GD}$	Variant II	0.0230	0.0187 (19%)	0.0089 (52%)	0.0076 (15%)	0.0072 (5%)	69%	60%
	Variant III	0.0221	0.0344 (−56%)	0.0226 (34%)	0.0227 (0%)	0.0182 (20%)	18%	—

## Data Availability

The original contributions presented in this study are included in the article. Further inquiries can be directed to the corresponding author.

## Abbreviations

The following abbreviations are used in this manuscript:

GA	Genetic algorithm
FEM	Finite Element Method
MF	Multi-fidelity
LF	Low-fidelity
HF	High-fidelity
CFRP	Carbon Fiber-Reinforced Polymer
GFRP	Glass Fiber-Reinforced Polymer
tFRP	Theoretical Fiber-Reinforced Polymer
NSGAII	Non-dominated Sorting Genetic Algorithm
DNN	Deep neural network
CL	Curriculum Learning
TPF	True Pareto Front

## References

[1] Nikbakt S., Kamarian S., Shakeri M. (2018). A review on optimization of composite structures Part I: Laminated composites. Compos. Struct..

[2] Ghiasi H., Pasini D., Lessard L. (2009). Optimum stacking sequence design of composite materials Part I: Constant stiffness design. Compos. Struct..

[3] Setoodeh S., Abdalla M.M., Gürdal Z. (2006). Design of variable–stiffness laminates using lamination parameters. Compos. Part Eng..

[4] Chiachio M., Chiachio J., Rus G. (2012). Reliability in composites—A selective review and survey of current development. Compos. Part Eng..

[5] Reddy J.N. (2004). Mechanics of Laminated Composite Plates and Shells: Theory and Analysis.

[6] Goldberg D.E. (1989). Genetic Algorithms in Search, Optimization and Machine Learning.

[7] Callahan K.J., Weeks G.E. (1992). Optimum design of composite laminates using genetic algorithms. Compos. Eng..

[8] Riche R.L., Haftka R.T. (1993). Optimization of laminate stacking sequence for buckling load maximization by genetic algorithm. AIAA J..

[9] Sivanandam S., Deepa S.N. (2008). Introduction to Genetic Algorithms.

[10] Wang G.G., Shan S. (2006). Review of Metamodeling Techniques in Support of Engineering Design Optimization. J. Mech. Des..

[11] Forrester A.I.J., Sóbester A., Keane A.J. (2008). Engineering Design via Surrogate Modelling.

[12] Forrester A.I., Keane A.J. (2009). Recent advances in surrogate-based optimization. Prog. Aerosp. Sci..

[13] Peherstorfer B., Willcox K., Gunzburger M. (2018). Survey of Multifidelity Methods in Uncertainty Propagation, Inference, and Optimization. SIAM Rev..

[14] Kleijnen J.P. (2009). Kriging metamodeling in simulation: A review. Eur. J. Oper. Res..

[15] Toal D.J.J. (2015). Some considerations regarding the use of multi-fidelity Kriging in the construction of surrogate models. Struct. Multidiscip. Optim..

[16] Dadras Eslamlou A., Huang S. (2022). Artificial-Neural-Network-Based Surrogate Models for Structural Health Monitoring of Civil Structures: A Literature Review. Buildings.

[17] Alizadeh R., Allen J.K., Mistree F. (2020). Managing computational complexity using surrogate models: A critical review. Res. Eng. Des..

[18] Zhang L., Choi S.K., Xie T., Jiang P., Hu J., Koo J. (2021). Multi-fidelity surrogate model-assisted fatigue analysis of welded joints. Struct. Multidiscip. Optim..

[19] Zhang X., Xie F., Ji T., Zhu Z., Zheng Y. (2021). Multi-fidelity deep neural network surrogate model for aerodynamic shape optimization. Comput. Methods Appl. Mech. Eng..

[20] Liao P., Song W., Du P., Zhao H. (2021). Multi-fidelity convolutional neural network surrogate model for aerodynamic optimization based on transfer learning. Phys. Fluids.

[21] Waszczyszyn Z., Ziemiański L. (2001). Neural Networks in Mechanics of Structures and Materials-New Results and Prospects of Applications. Comput. Struct..

[22] LeCun Y., Bengio Y., Hinton G. (2015). Deep learning. Nature.

[23] Goodfellow I., Bengio Y., Courville A. (2016). Deep Learning.

[24] Bengio Y., Louradour J., Collobert R., Weston J. Curriculum learning. Proceedings of the 26th Annual International Conference on Machine Learning.

[25] Hsiao Y.D., Chang C.T. (2023). Progressive learning for surrogate modeling of amine scrubbing CO2 capture processes. Chem. Eng. Res. Des..

[26] Fayek H.M., Cavedon L., Wu H.R. (2020). Progressive learning: A deep learning framework for continual learning. Neural Netw..

[27] Kong Y., Liu L., Wang J., Tao D. Adaptive Curriculum Learning. Proceedings of the 2021 IEEE/CVF International Conference on Computer Vision (ICCV).

[28] Scherding C., Rigas G., Sipp D., Schmid P.J., Sayadi T. (2025). An adaptive learning strategy for surrogate modeling of high-dimensional functions—Application to unsteady hypersonic flows in chemical nonequilibrium. Comput. Phys. Commun..

[29] Riquelme N., Lückenand C.V., Baran B. Performance metrics in multi-objective optimization. Proceedings of the 2015 Latin American Computing Conference (CLEI).

[30] Audet C., Bigeon J., Cartier D., Le Digabel S., Salomon L. (2021). Performance indicators in multiobjective optimization. Eur. J. Oper. Res..

[31] Queipo N.V., Haftka R.T., Shyy W., Goel T., Vaidyanathan R., Kevin Tucker P. (2005). Surrogate-based analysis and optimization. Prog. Aerosp. Sci..

[32] Hwang J.T., Martins J.R. (2018). A fast-prediction surrogate model for large datasets. Aerosp. Sci. Technol..

[33] Jing Z., Sun Q., Zhang Y., Liang K., Li X. (2021). Stacking sequence optimization of doubly-curved laminated composite shallow shells for maximum fundamental frequency by sequential permutation search algorithm. Comput. Struct..

[34] Chen Y., Wang Q., Zhong R., Shi X., Qin B. (2023). Fiber orientation and boundary stiffness optimization of laminated cylindrical shells with elastic boundary for maximum the fundamental frequency by an improved sparrow search algorithm. Thin-Walled Struct..

[35] Chaudhuri P.B., Mitra A., Sahoo S. (2023). Maximization of Fundamental Frequency of Composite Stiffened Hypar Shell with Cutout by Taguchi Method. Mech. Adv. Compos. Struct..

[36] Serhat G. (2021). Design of Circular Composite Cylinders for Optimal Natural Frequencies. Materials.

[37] Alshabatat N.T. (2022). Natural Frequencies Optimization of Thin-Walled Circular Cylindrical Shells Using Axially Functionally Graded Materials. Materials.

[38] Chen D., Sun G., Meng M., Jin X., Li Q. (2019). Flexural performance and cost efficiency of carbon/basalt/glass hybrid FRP composite laminates. Thin-Walled Struct..

[39] Bathe K.J. (2016). ADINA: Theory and Modeling Guide Volume I: ADINA Solids & Structures.

[40] Miller B., Ziemiański L. (2020). Optimization of dynamic behavior of thin-walled laminated cylindrical shells by genetic algorithms and deep neural networks supported by modal shape identification. Adv. Eng. Softw..

[41] Deb K., Pratap A., Agarwal S., Meyarivan T. (2002). A fast and elitist multiobjective genetic algorithm: NSGA-II. IEEE Trans. Evol. Comput..

[42] Miller B., Ziemiański L. (2023). Multi-Objective Optimization of Thin-Walled Composite Axisymmetric Structures Using Neural Surrogate Models and Genetic Algorithms. Materials.

[43] Miller B., Ziemiański L. (2024). Optimizing composite shell with neural network surrogate models and genetic algorithms: Balancing efficiency and fidelity. Adv. Eng. Softw..

[44] (2019). Mathematica.

[45] Ahn J.G., Yang H.I., Kim J.G. (2022). Multi-fidelity meta modeling using composite neural network with online adaptive basis technique. Comput. Methods Appl. Mech. Eng..

[46] Guo M., Manzoni A., Amendt M., Conti P., Hesthaven J.S. (2022). Multi-fidelity regression using artificial neural networks: Efficient approximation of parameter-dependent output quantities. Comput. Methods Appl. Mech. Eng..

[47] Tan J., Shao Y., Zhang J., Zhang J. (2024). Efficient Antenna Modeling and Optimization Using Multifidelity Stacked Neural Network. IEEE Trans. Antennas Propag..

[48] Tian Y., Cheng R., Zhang X., Li M., Jin Y. (2019). Diversity Assessment of Multi-Objective Evolutionary Algorithms: Performance Metric and Benchmark Problems [Research Frontier]. IEEE Comput. Intell. Mag..

[49] Zitzler E., Thiele L., Laumanns M., Fonseca C.M., da Fonseca V.G. (2003). Performance assessment of multiobjective optimizers: An analysis and review. IEEE Trans. Evol. Comput..

[50] Zitzler E., Deb K., Thiele L. (2000). Comparison of Multiobjective Evolutionary Algorithms: Empirical Results. Evol. Comput..

[51] Vargas A., Bogoya J. (2018). A Generalization of the Averaged Hausdorff Distance. Comput. Sist..

[52] Miller B., Ziemiański L. (2021). Identification of Mode Shapes of a Composite Cylinder Using Convolutional Neural Networks. Materials.

